# MetaSel: a metaphase selection tool using a Gaussian-based classification technique

**DOI:** 10.1186/1471-2105-14-S16-S13

**Published:** 2013-10-22

**Authors:** Ravi Uttamatanin, Peerapol Yuvapoositanon, Apichart Intarapanich, Saowaluck Kaewkamnerd, Ratsapan Phuksaritanon, Anunchai Assawamakin, Sissades Tongsima

**Affiliations:** 1 The Electrical Engineering Graduate Program, Faculty of Engineering, Mahanakorn University of Technology, Bangkok, Thailand; 2 Electronic Engineering Department Mahanakorn University of Technology, Bangkok, Thailand; 3 National Electronics and Computer Technology Center, Thailand Science Park, Pathum Thani, Thailand; 4 Food and Drug Administration Ministry of Public Health, Nonthaburi, Thailand; 5 Department of Pharmacology, Faculty of Pharmacy, Mahidol University, Bangkok, Thailand; 6 National Center for Genetic Engineering and Biotechnology, Thailand Science Park, Pathum Thani, Thailand

**Keywords:** Karyotype software, Chromosome, Metaphase selection, Metaphase spread, Rule-based classification, Gaussian model

## Abstract

**Background:**

Identification of good metaphase spreads is an important step in chromosome analysis for identifying individuals with genetic disorders. The process of finding suitable metaphase chromosomes for accurate clinical analysis is, however, very time consuming since they are selected manually. The selection of suitable metaphase chromosome spreads thus represents a major bottleneck for conventional cytogenetic analysis. Although many algorithms have been developed for karyotyping, none have adequately addressed the critical bottleneck of selecting suitable chromosome spreads. In this paper, we present a software tool that uses a simple rule-based system to efficiently identify metaphase spreads suitable for karyotyping.

**Results:**

The chromosome shapes can be classified by the software into four main classes. The first and the second classes refer to individual chromosomes with straight and skewed shapes, respectively. The third class is characterized as those chromosomes with overlapping bodies and the fourth class is for the non-chromosome objects. Good metaphase spreads should largely contain chromosomes of the first and the second classes, while the third class should be kept minimal. Several image parameters were examined and used for creating rule-based classification. The threshold value for each parameter is determined using a statistical model. We observed that the Gaussian model can represent the empirical probability density function of the parameters and, hence, the threshold value can be easily determined. The proposed rules can efficiently and accurately classify the individual chromosome with > 90% accuracy.

**Conclusions:**

The software tool, termed MetaSel, was developed. Using the Gaussian-based rules, the tool can be used to quickly rank hundreds of chromosome spread images so as to assist cytogeneticists to perform karyotyping effectively. Furthermore, MetaSel offers an intuitive, yet comprehensive, workflow to assist karyotyping, including tools for editing chromosome (split, merge and fix) and a karyotyping editor (moving, rotating, and pairing homologous chromosomes). The program can be freely downloaded from "http://www4a.biotec.or.th/GI/tools/metasel".

## Background

**Figure 1:**
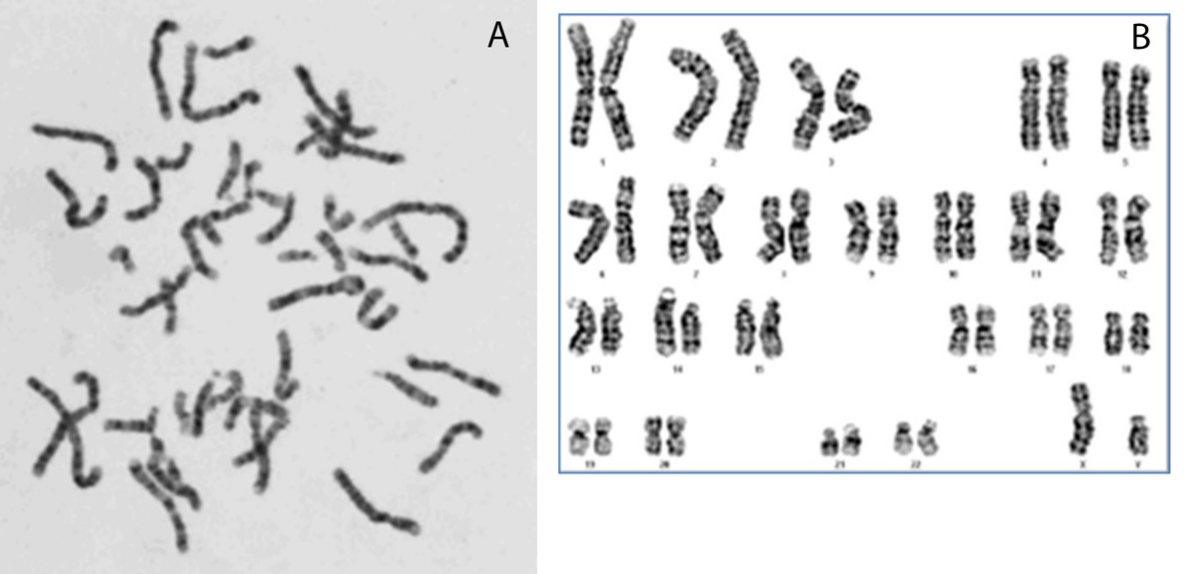
**Metaphase image and Karyotyping image**. (A) The metaphase image with good metaphase spread that is proper for karyotyping (B) The karyotyping image with ordered/labelled chromosomes.

In cytogenetic studies, abnormalities in chromosome structure are examined by microscopy. Each human cell normally has 23 pairs of chromosomes, consisting of 22 pairs of autosomes and one pair of sex chromosomes [1,2]. Cytogenetic abnormalities are manifested as extra or fewer chromosomes than normal, e.g., having three copies of chromosome 21 in Down's syndrome, one of the most common abnormalities. Cytogenetic testing for abnormalities requires high-quality metaphase chromosome images, which are selected and sorted as shown in Figure 1.

In order to obtain enough analyzable metaphase spread images, at least 8 to 10 glass slide specimens have to be prepared for each individual. Each glass slide typically contains about 10-20 metaphase spreads. From the total of approximately 200 prepared metaphases, approximately 20 of the "best" (based on the subjective opinion of an experienced cytogeneticist) metaphase spreads are selected for karyotyping [1].

The consistency of chromosome numbers, i.e. total chromosome complement of each cell, is commonly determined by visual inspection among these top twenty metaphase spreads. Once the chromosome complement is verified, generally two to five of the "sharpest" images are chosen for chromosome banding analysis for detecting chromosome band abnormalities. Each step in this process is time consuming and requires experienced cytogeneticists to operate. Thus, considerable effort has been made to develop automated chromosome image analysis tools to expedite this procedure.

Each metaphase spread contains not only chromosome images but also some cell preparation artifacts [1-5]. These non-chromosome residues can be eliminated by visual inspection. However, in order to obtain an accurate karyotyping result, the metaphase spread must contain a large number of analyzable chromosomes, i.e., with clear banding patterns not obscured by overlapping chromosomes. Previous research efforts have mainly focused on segmentation of overlapping chromosomes [1,6,7]. However, when overlapping chromosome images are segmented, the regions of chromosome overlap are ambiguous, which could potentially lead to an inaccurate diagnosis. Therefore, getting clean metaphase spreads with well-separated individual chromosomes is preferable.

Other earlier studies on chromosome analysis have concentrated on automatic karyotyping which attempts to order and classify the chromosomes into 22 pairs of autosomes and the two sex chromosomes. Automatic karyotyping requires very informative features, such as band profiles, centromere positions, chromosome dimensions, etc. Automatic karyotyping is based on the assumption that the input contains analyzable metaphases. Numerous algorithms have been proposed to facilitate automatic karyotyping [4-7]. A recent technique proposed by Moallem et al. [17] used dark paths between chromosomes for classifying touching and overlapping chromosomes from good metaphase images. Khan et al. [18] presented a technique to geometrically correct deformed chromosomes so that the chromosomes can be karyotyped correctly. Jahani et al [19] focused on classification by identifying chromosome centromeres and their corresponding length.

To perform automatic karyotyping, hundreds of images must be manually examined in order to select spreads comprising mostly metaphase chromosomes for further analysis. The goal is thus to select the best metaphase spreads with clearly separated individual chromosomes for karyotyping. The selection of good, metaphase spreads is very time consuming, perhaps requiring hours of expert inspection of hundreds of specimens. Thus, the cytogeneticist will normally select approximately 20 of the *first *good metaphase spreads that he/she has encountered, instead of examining all metaphase spreads from all specimen slides. Hence, this arbitrary approach may exclude better metaphase spreads, and so lead to sub-optimal results. There is thus a need for a more thorough and efficient method of selecting good metaphase spreads for karyotyping. Although some techniques have been proposed for automatic metaphase selection, in practice these techniques are impractical for processing hundreds of images in a typical cytogenetic analysis owing to the high computational complexity [1-5]; [13-15].

To our knowledge, there are only two works that have addressed the problem of improving the efficiency of automated metaphase selection. The first study [12] concentrated on rapid identification of metaphase, but did not assess metaphase *quality*, i.e. the selection of analyzable versus non-analyzable metaphase. The second approach in [9] utilizes skeletal analysis of chromosome images in order to estimate the number of analyzable chromosomes; hence, it can quickly select a few good metaphase spreads in terms of quality. However, the time to process each image can take up to 5 minutes, which is still not practical when dealing with a large number (>100) of images.

To address the aforementioned problems, this work presents a rapid, practical chromosome classification tool for identification of good metaphase spreads based on rule-based classification. The software, called MetaSel, is the first attempt to offer a free assistive karyotyping tool for chromosome analysis. The software employs a heuristic that first defines important image parameters for chromosome feature extraction and then constructs rules for chromosome classification.

### Materials and methods

#### Overview

**Figure 2:**
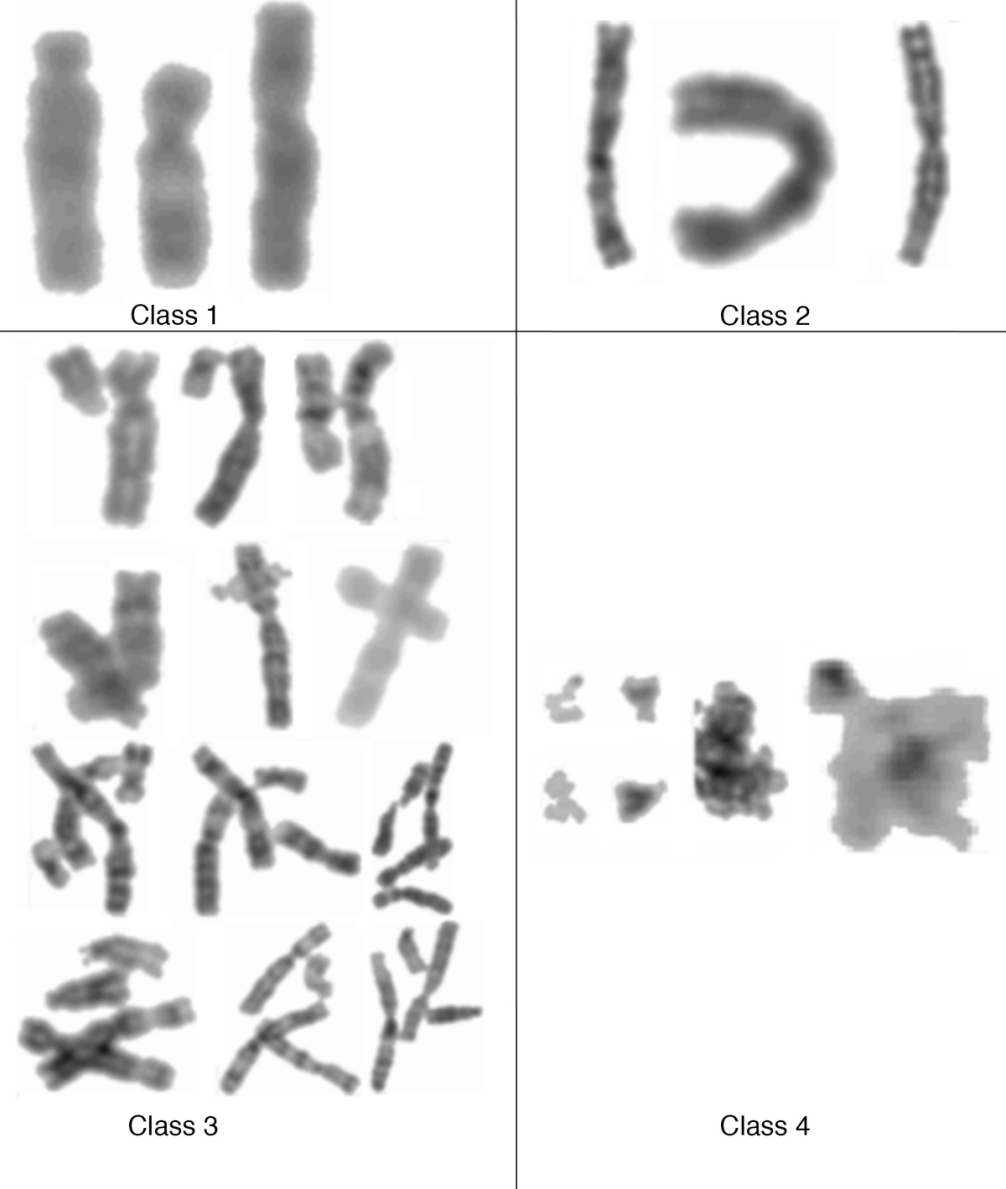
**Types of chromosome classification**. Chromosomes from Class-1 and Class-2 are individually separated. Both classes are differentiated by their straightness, i.e., Class-1 is straight individual chromosome while Class-2 is individually separable but with bended or skewed structure. Class-3 chromosomes are those that appear touching/overlapping with other chromosomes. Finally, Class-4 is characterized as non-chromosome residues and to be excluded in future analyses.

Specimens for cytogenetic testing were obtained by a standard clinical procedure at the Rajanukul Institute, Ministry of Public Health, Bangkok. In brief, cells from amniocentesis samples from pregnant women were applied to glass slides and stained with Giemsa. Chromosome images were obtained by microscopy using the Zeiss Axioskop2 model. A metaphase spread contains some individual chromosomes as well as other chromosomes that may not be well spread out, i.e., overlapping or touching. We defined objects from the metaphase spreads into four classes (Figure 2). The first three classes are in fact the underlying chromosomes whereas Class-4 is considered as residues or artifacts, e.g., cell debris. Individual chromosomes from Class-1 and Class-2 can be distinguished by their straightness. Chromosomes from both classes must be individually separable. Hence, Class-1 is defined as straight individual chromosome, while Class-2 is defined as skewed or bended individual chromosome. Chromosomes from Class-3 comprise other non-individual chromosomes that may be overlapping or touching with other chromosomes in the vicinity.

#### Pre-processing

First an image is enhanced by using the histogram equalization threshold as described in [10,11] for adjusting the gray level in the image. Then, we attempted to separate the real chromosome image from its background. This process is called image segmentation in image processing [16]. In order to do the segmentation, we adopted the Otsu's automatic threshold technique [8] to isolate the chromosome image from the background.

#### Chromosome classification

**Figure 3:**
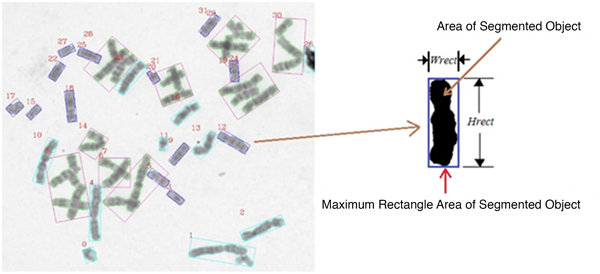
**Image parameters for chromosome image classification**. The segmented chromosomal objects in the left metaphase image are rotated into vertical orientation and calculated image parameters. *W_rect _*and *H_rect _*are width (pixel) and height (pixel) of the smallest enclosing rectangle of the segmented object respectively.

We performed image segmentation and rotated the resulting objects into their vertical orientation in order to classify segmented objects from metaphase spreads. The image parameters, namely width, height, and estimated area ratio, are extracted from the rotated images. The width and height parameters of each chromosome segment are the important factors used to quickly characterize the chromosomal objects into the four classifications. In particular, the area ratio can be defined as:

$$Area_{ratio}=\frac{A_{o}}{A_{r}}$$

where *A_r _*is the number of pixels inside the smallest enclosing rectangle (*W_rect _×H_rect_*) of the segmented object and *A_o _*is the number of pixels of the segmented object. Figure 3 shows image parameters for chromosome image classification, where *W_rect _*and *H_rect _*are the width and the height of the minimum rectangle of segmented objects in pixel unit.

**Figure 4:**
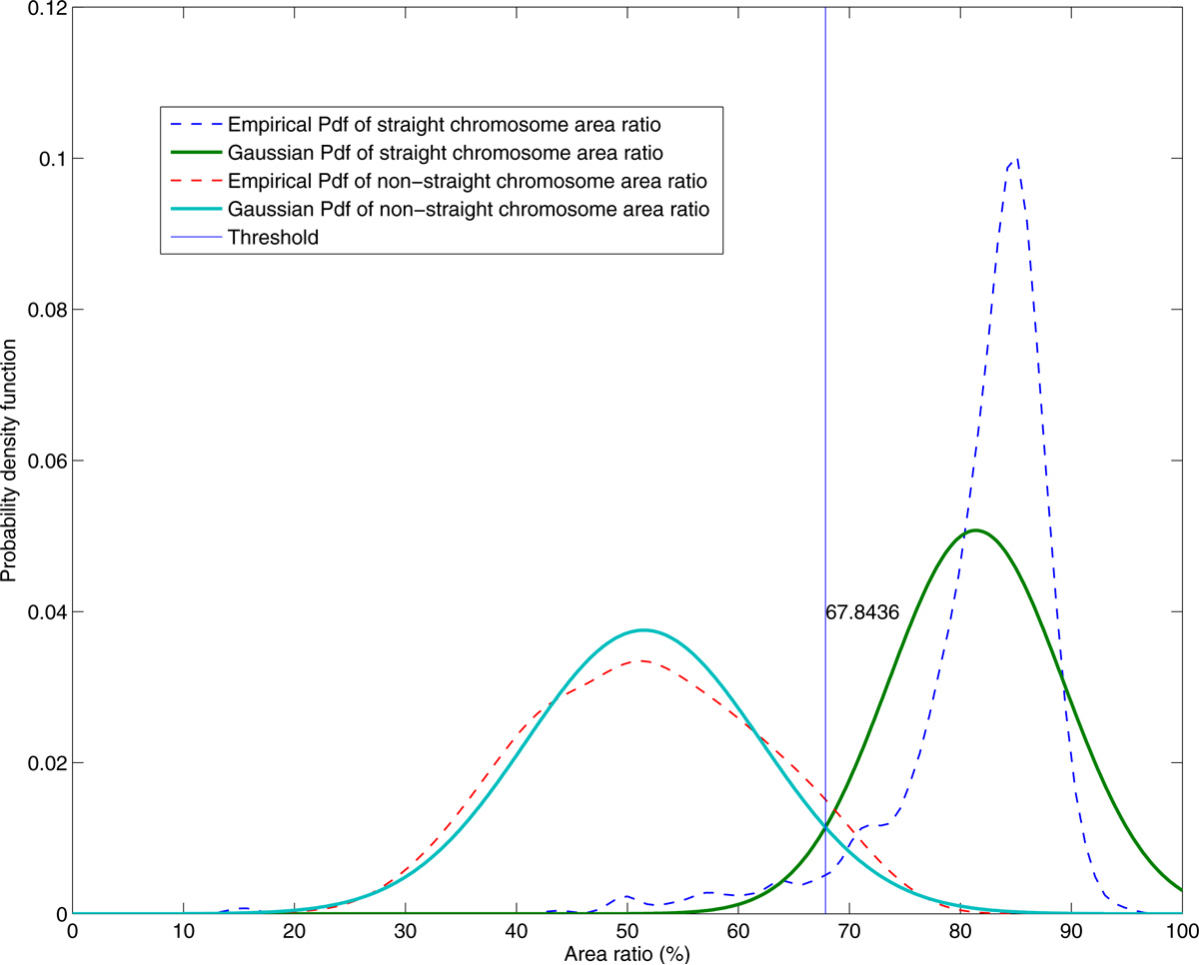
**Empirical and Gaussian probability density functions of the area ratio**. Gaussian model was used to determine the threshold value of the area ratio for classification. When the area ratio is greater than 67.84%, the chromosome can be classified as Class-1, straight object.

The area ratio quantifies the amount of the actual object pixels per the pixels inside the rectangle box demarcating the object. This ratio can be *effectively *used to classify the straightness of the chromosome. We verified this ratio by performing statistical analysis of randomly chosen chromosome area ratios from 822 straight and 1012 touching/overlapping (including skewed objects) chromosomes. The empirical probability density function was estimated using the kernel density method (Figure 4). Gaussian model was used to determine the threshold value of the area ratio for classification. When the area ratio is greater than 67.84%, the chromosome can be classified as Class-1 (straight objects). However, this class may contain some non-chromosome residues that need to be excluded.

Since the width of Class-1 chromosomes should be consistent, deviation from their average width is considered as residual objects. To detect these remainders, we first determine the total average width of all objects with the area ratio > 67.84%. If the object width is greater than 1.5 times of the total average width, such an object will be discarded. Let *O*_w _represent the set of objects with the underlying width less than 1.5 times of the total average width. The chromosome width of each object (*W*) in the set *O*_w _can be defined as:

$$W_{i}=\frac{Total\;number\;of\;pixels\;in\;chromosome}{H_{rect}}$$

Then, the average width is defined as:

$$W_{avg}=\frac{\underset{i\in O_{W}}{\sum }W_{i}}{\left|O_{W}\right|}$$

To quantify the deviation from the average width, we define the rectangle width ratio as:

$$Wrect_{ratio}=\frac{W_{rect}}{W_{avg}}$$

**Figure 5:**
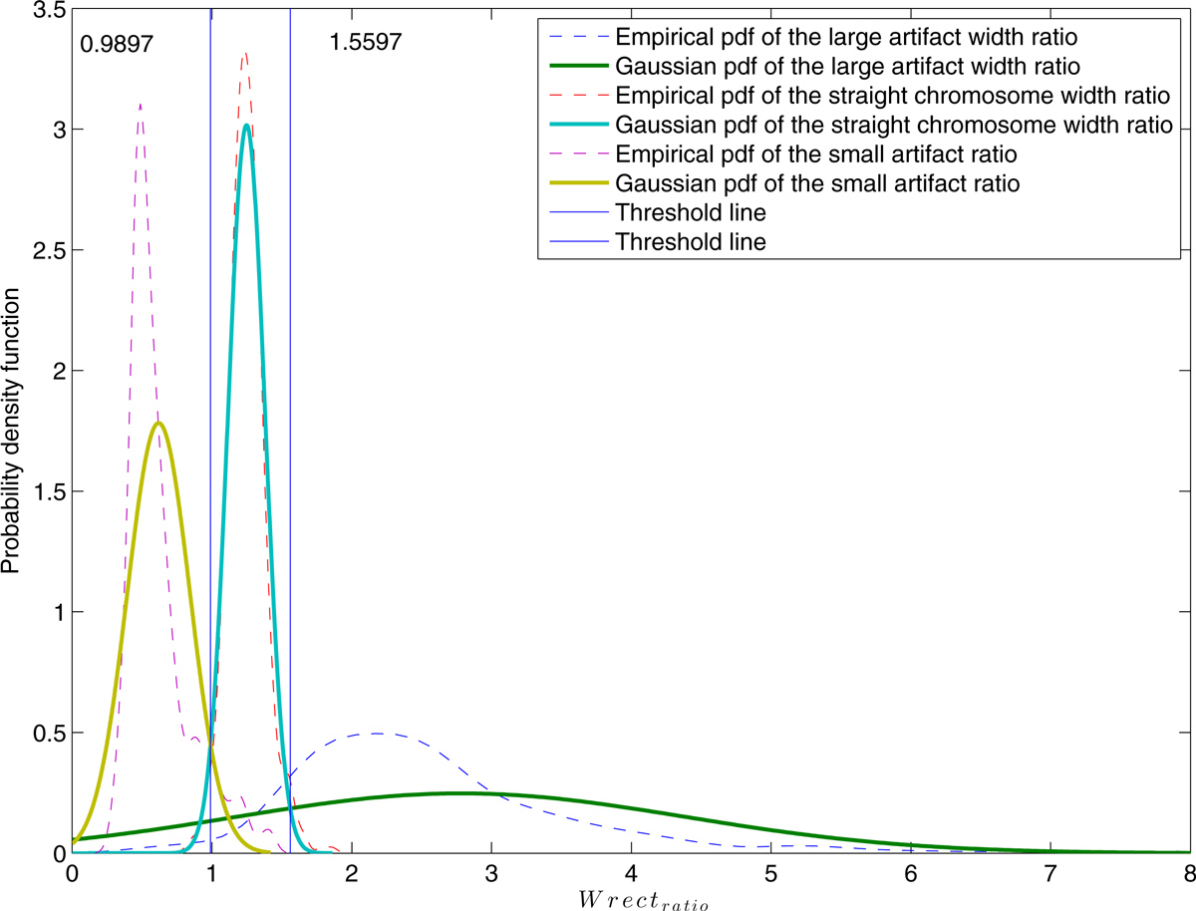
**Empirical and Gaussian probability density functions of the *Wrect_ratio. _***The experimental studies of this ratio were performed using 222, 327 and 500 samples of small, large residual objects and straight individual chromosomes respectively. When 0.9897 ≤ *Wrect_ratio _*≤ 1.5597, the object is classified as straight individual chromosome (Class-1) while if *Wrect_ratio _*<0.9897 indicates that the object is potentially a small non-chromosome residue (Class-4). The object is considered to be a large residue (Class-4) when *Wrect_ratio _*> 1.5597.

Clearly, the deviation from the unity of *Wrect_ratio _*entails differences in terms of the quality of chromosome straightness. Thus, the threshold value of the rectangle width ratio for Class-1 is determined by the probability distribution of *Wrect_ratio_*. The experimental studies of this ratio were performed using 222, 327 and 500 samples of small, large residual objects and straight individual chromosomes respectively. The empirical and Gaussian probability density functions of *Wrect_ratio _*are depicted in Figure 5. When 0.9897 ≤ *Wrect_ratio _*≤ 1.5597, the corresponding object will be classified as straight individual chromosome (Class-1). When *Wrect_ratio _*<0.9897, the chromosome object will be classified as a small non-chromosome residue (Class-4). Moreover, the object can be classified as Class-4 when *Wrect_ratio _*> 1.5597, i.e., being a large object.

When *Wrect_ratio _*< 67.84%, the corresponding object can be classified as either skewed individual chromosome or touching/overlapping chromosome. To distinguish between skewed objects and non-chromosome residues, the height of segmented object is defined as:

$$H_{i}=\frac{A_{o}}{W_{avg}}$$

The ratio between *H_i _*and *H_rect_*, height ratio (*Hi_ratio_*), is computed by.

$$Hi_{ratio}=\frac{H_{i}}{H_{rect}}$$

**Figure 6:**
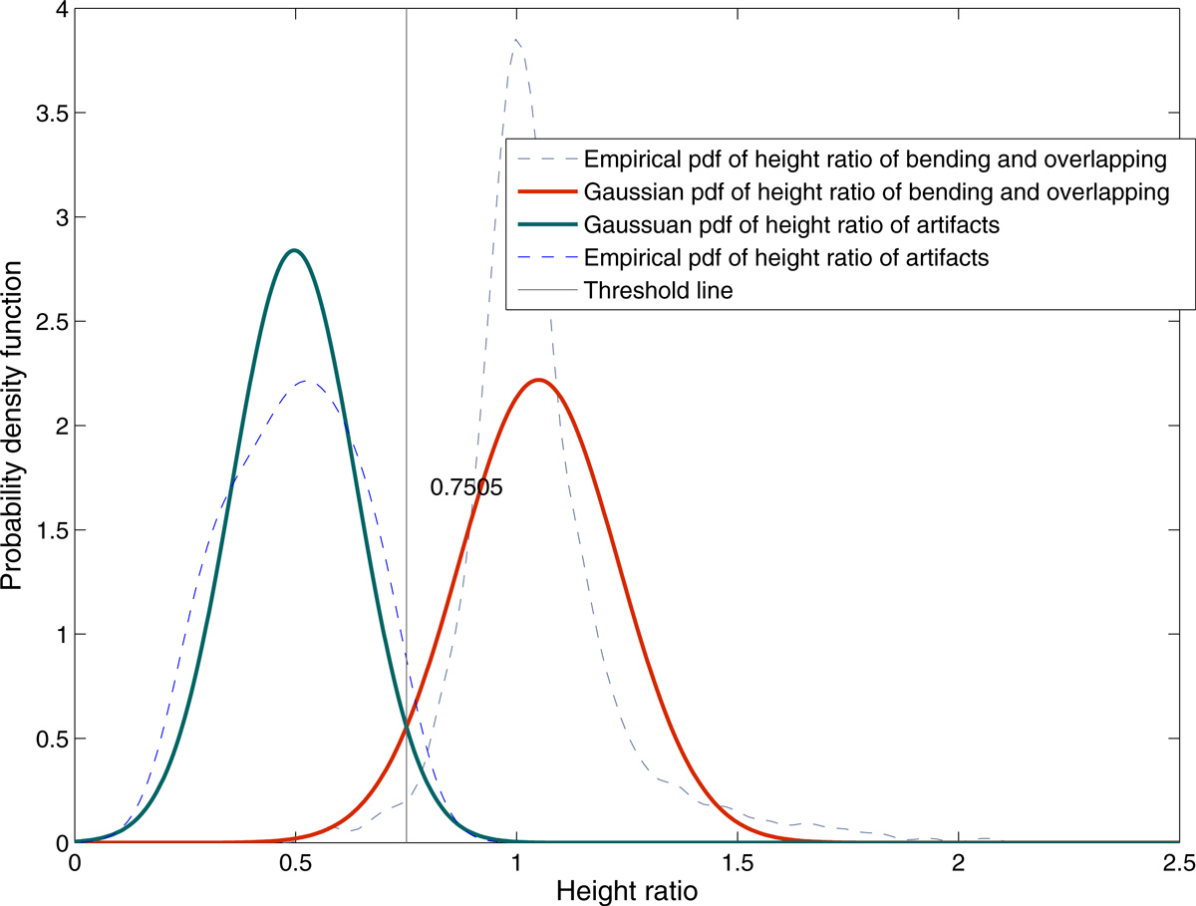
**Empirical and Gaussian probability density functions of the height ratio**. The statistical analysis was performed to determine the threshold value of the height ratio for eliminating residual objects. It can be observed that the empirical probability density function can be approximated by Gaussian model. From the Gaussian model, the objects are classified as residual objects when *Hi_ratio _*< 0.7507. When *Hi_ratio _*≥ 0.7507, the objects are classified as mixing between skewed objects and touching/overlapping chromosomes.

We observed 600 skewed objects and overlapping chromosomes as well as 70 non-chromosome residues. The statistical analysis was performed to determine the threshold value of the height ratio for screening out unwanted residual objects. Figure 6 presents the empirical probability density function of the height ratio which can be approximated by the Gaussian model. Using this model, chromosome objects will be classified as "residual" when *Hi_ratio _*< 0.7507. When *Hi*_ratio _≥ 0.7507, the objects will be classified as mixing between skewed objects and touching/overlapping chromosomes.

To separate skewed objects from those touching/overlapping chromosomes, one additional parameter must be used. It can be observed that the width of an overlapping chromosome will be larger than the width of a skewed individual. This parameter, called maximum width ratio (*W*max*_ratio_*), therefore, can be computed by using the maximum object width in pixels (*W*max) and the average width (*W*_avg_):

$$W{\text{max}}_{ratio}=\frac{W_{\text{max}}}{W_{avg}}$$

**Figure 7:**
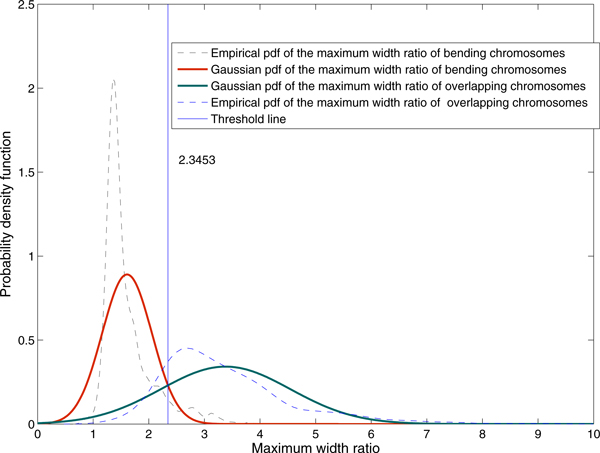
**Empirical and Gaussian probability density functions of the maximum width ratio**. Gaussian model was used to approximate the empirical model for threshold calculation. The threshold for separating skewed individuals and overlapping chromosomes was chosen to be 2.3453 (the intercept between the two Gaussian curves). In other words, the objects will be classified as overlapping chromosomes when *W*max*_ratio _*is greater than this selected threshold. When *W*max*_ratio _*is less than or equal to the threshold, objects will be classified as skewed individuals.

**Figure 8:**
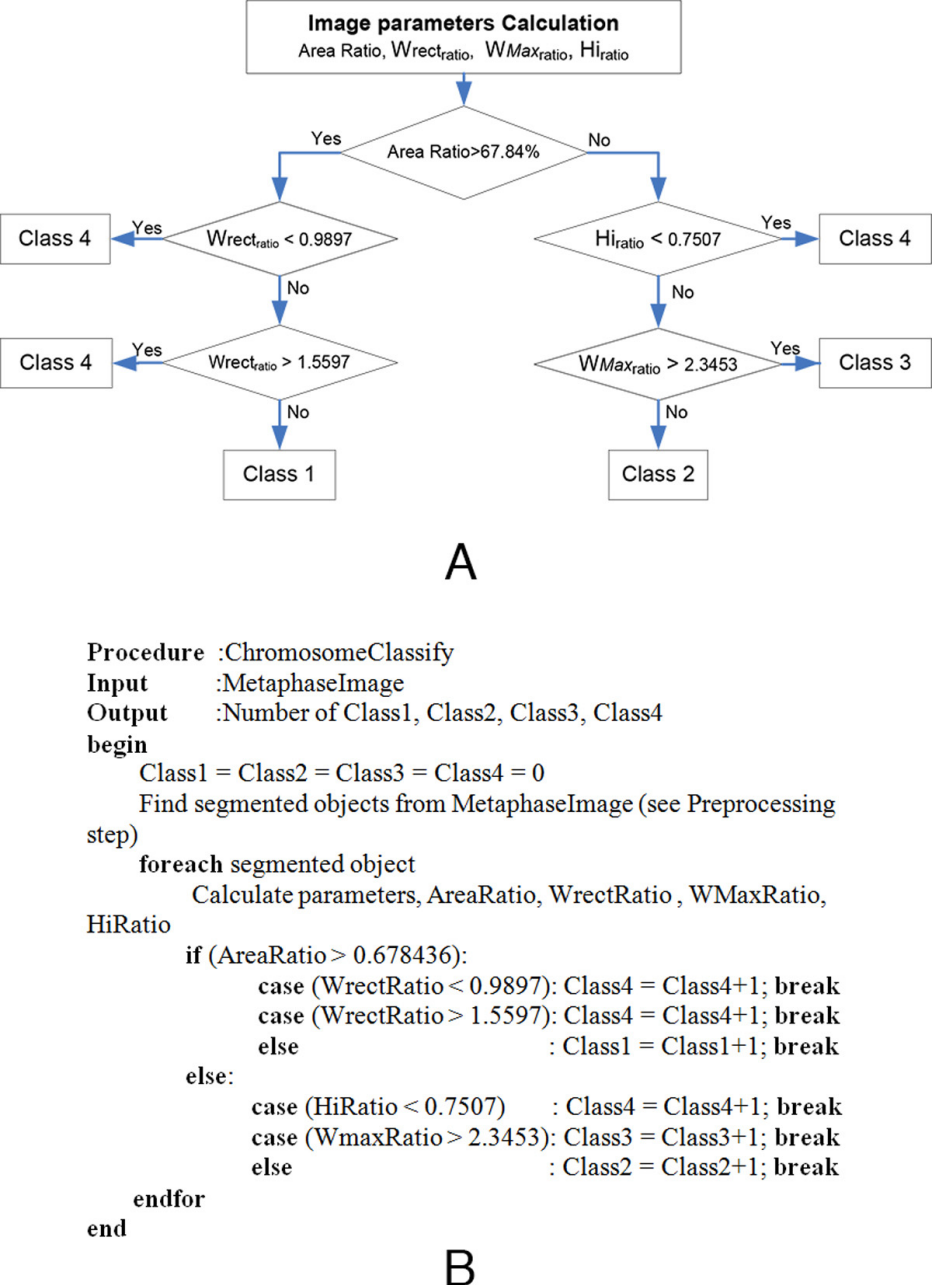
**Chromosomal image classification rule**. (A) The proposed decision rule is used to classify chromosome images into four classes, straight individual chromosome (Class-1), skewed individual chromosome (Class-2), touching/overlapping chromosomes (Class-3) and non-chromosome residues (Class-4). (B) Pseudo-code of the previous rule-based flowchart.

The threshold to separate skewed chromosome individuals from overlapping chromosomes was determined by using statistical analysis. The empirical probability density functions of skewed individuals and overlapping chromosomes were determined using 593 and 393 samples respectively. The Gaussian model was used to approximate the empirical model for threshold calculation. The threshold for separating skewed individuals and overlapping chromosomes was chosen to be the intercept of the two Gaussian curves (2.3453) as shown in Figure 7. In other words, the objects will be classified as overlapping chromosomes when *W*max*_ratio _*is greater than this selected threshold. When *W*max*_ratio _*is less than or equal to the threshold, objects will be classified as skewed individuals. Figure 8 summarizes image parameters (see flowchart in panel A) and the proposed rule-based algorithm (see panel B) to classify chromosome images.

#### Implementation of MetaSel

**Figure 9:**
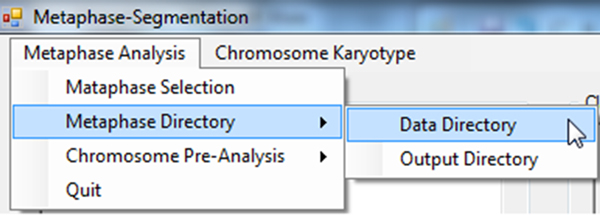
**Demonstration: Selecting metaphase image input directory**. Users first choose the input directory containing metaphase images (Metaphase Analysis →Metaphase Directory tab).

**Figure 10:**
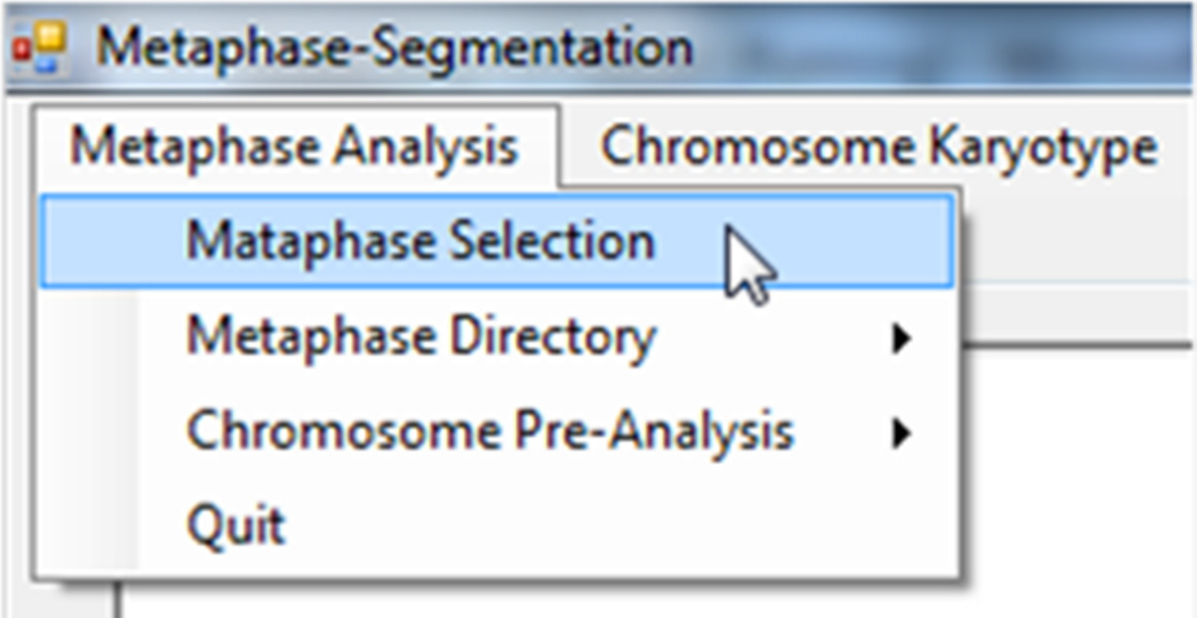
**Demonstration: Performing metaphase selection**. After choosing the input directory, users can click the menu "Metaphase Selection" to perform the metaphase selection process.

**Figure 11:**
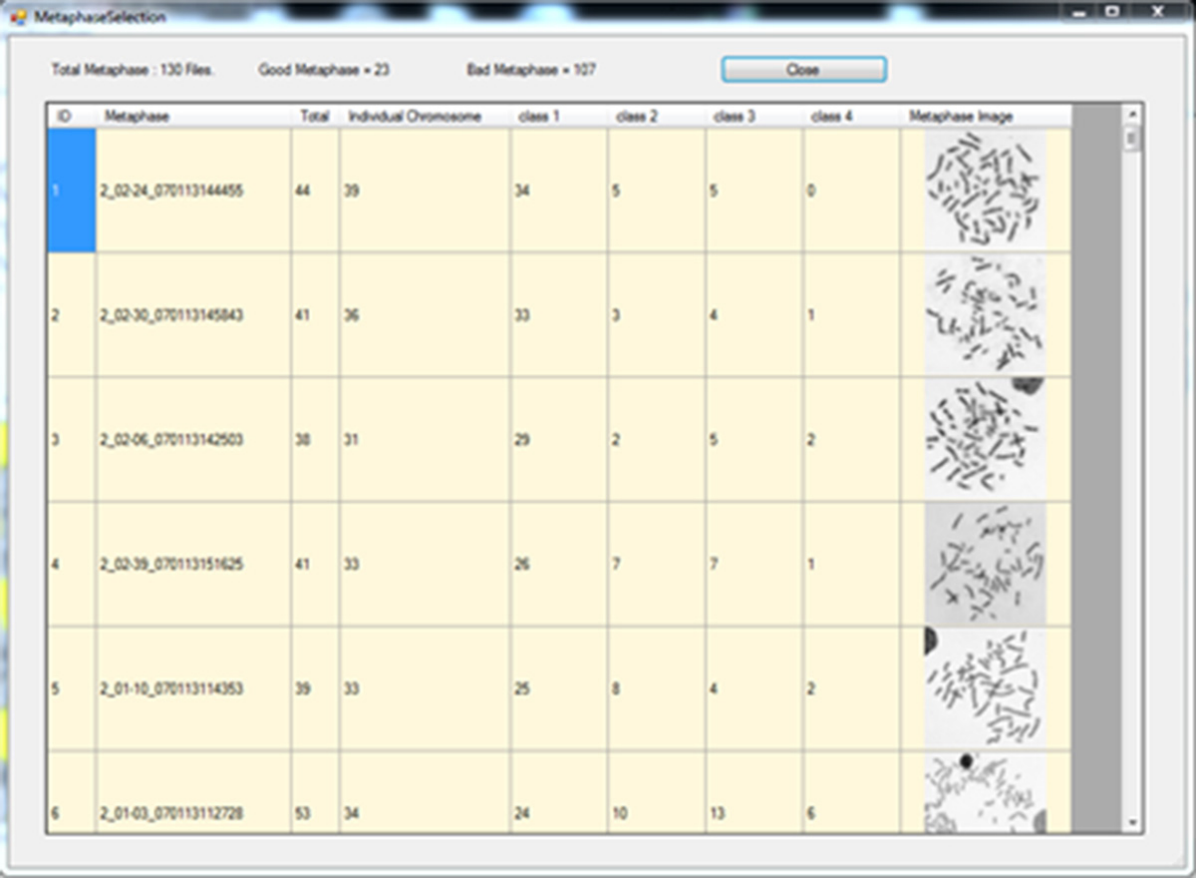
**Demonstration: window showing metaphase selection output**. The metaphase images will be grouped into four classes and ranked according to the total number of individual chromosomes, which is calculated by combining the number of objects in Class-1 and Class-2.

**Figure 12:**
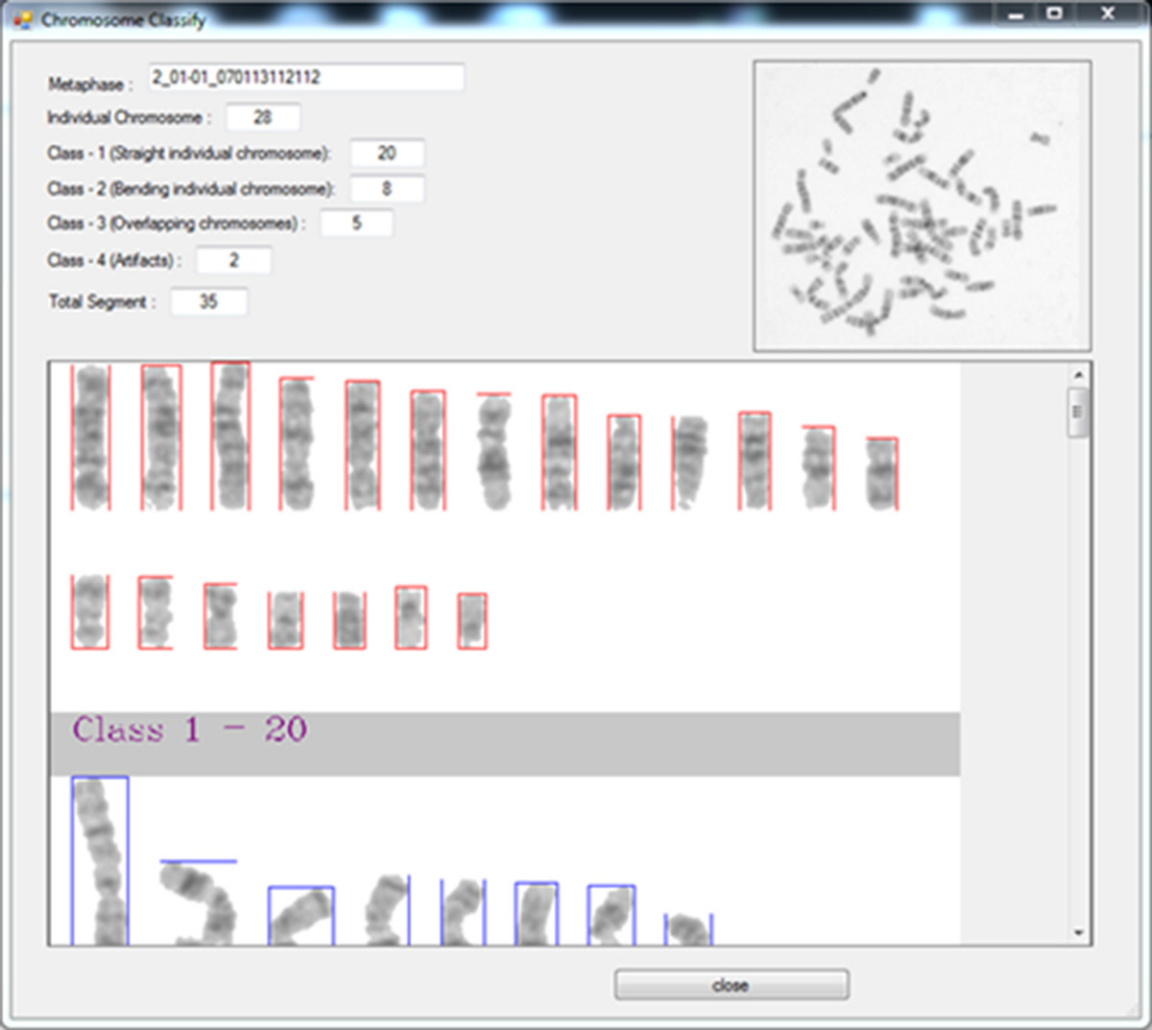
**Demonstration: Classification results of input Metaphase spreads**. MetaSel lined up the individual chromosomes from Class-1, and Class-2. Users can observe the detail of the metaphase images and choose the analyzable image to perform karyotyping.

**Figure 13:**
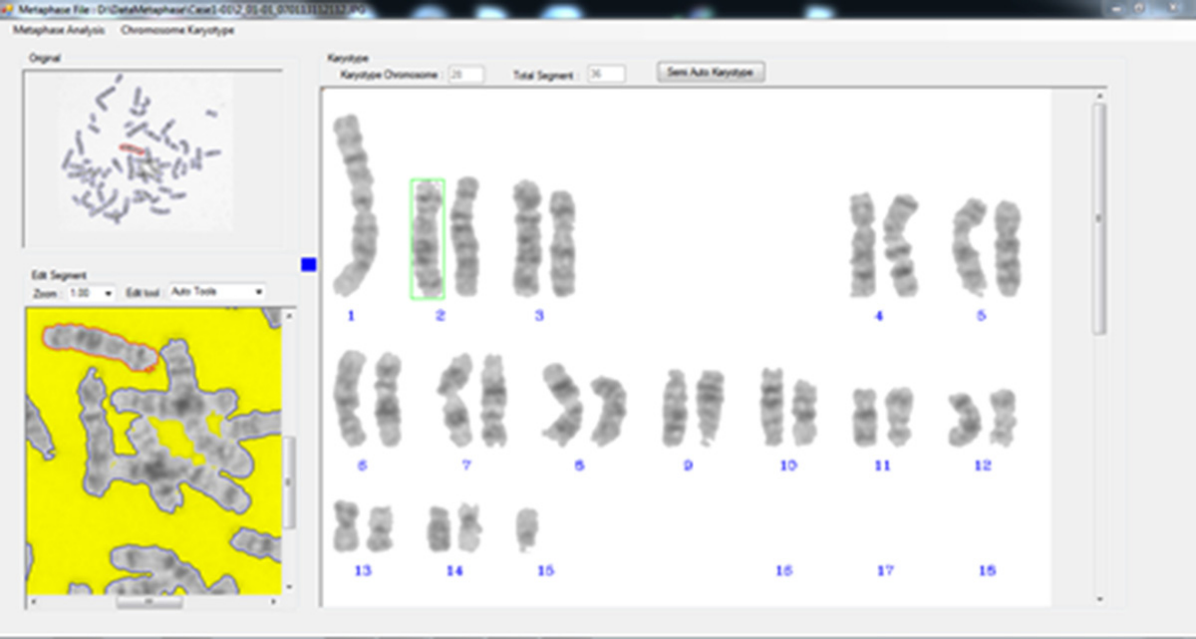
**Demonstration: Semi-automatic karyotyping**. MetaSel arranged all individual chromosomes by their lengths automatically. The touching/overlapping chromosomes were manually edited to separate them into individual chromosomes before karyotyping.

The proposed rule-based classification for metaphase selection was implemented in C# with OpenCV library. This classification module was incorporated into our karyotyping software tool, called MetaSel, which was written from scratch using C# on Microsoft Windows 7 operating system. Based on the decision rules presented in Figure 8, the workflow of this tool can be described as follows:

1. Open a project folder, which contains metaphase spread images (Figure 9).

2. Performing metaphase analysis by using the proposed classification rule (Figure 10).

3. The metaphase images will be grouped into four classes and ranked according to their total number of individual chromosomes, which is calculated by combining the number of objects in Class-1 and Class-2 (Figure 11).

4. Users choose which metaphase spread image to perform karyotyping. The higher rank generally refers to better quality (analyzable) of the spread. In case of a tie, users are strongly advised to choose the image that contains more objects in Class-3. If the number of objects in Class-3 is equal for the tie images, the number of object in Class-4 (smaller is better) should be used to break the tie.

5. After choosing the metaphase spread image, MetaSel will line up the individual chromosomes from Class-1, and Class-2 (Figure 12). Users can select good metaphase images to later perform karyotyping.

6. Users can go back to the original image to edit the ambiguous chromosome images (touching/overlapping objects) by cutting, merging, or fixing (make a correction on the contour line of a chromosome image), the images so that they can be karyotyped as described in the previous step. (Figure 13)

### Results

Two hundred metaphase spreads were used to determine the accuracy of the proposed rules. From these 192 metaphase images, 7817-segmented objects were obtained. The processing time for 192 metaphase images was 35.52 seconds and, hence, the average processing time for each image was approximately 0.185 seconds. The accuracy of this classification rule is shown in Table 1. We observe that only 0.58% of Class-1 was misclassified into Class-4. This classification error occurs due to residual objects that come with straight shape. Skewed individuals (Class-2) were misclassified as overlapping chromosomes (Class-3) or residual objects (Class-4). The accuracy of skewed individuals (Class-2) classification was 90.67%. Some of class-2 objects were classified into Class-3 and Class-4. This is because some overlapping chromosome arrangements were similar to the banding shape and some medium size residual objects. The accuracy of overlapping chromosomes (Class-3) classification is 89.44%. Some overlapping chromosomes are misclassified into Class-1, Class-2, and Class-3 since the random arrangements of overlapping pattern may resemble those classes. The rule gives very high accuracy (93.25%) of non-chromosome objects (Class-4) classification. There are only few percent of Class-4 misclassification.

**Table 1:** Classification Accuracy

Class	1	2	3	4
1	***5,103(99.42%)***	0	3 (0.30%)	36(0.48%)

2	0	***1,157(90.67%)***	92(9.26%)	34(2.54%)

3	0	99(7.76%)	***889(89.44%)***	37(2.50%)

4	30(0.58%)	20(1.57%)	10(1.01%)	***307(93.25%)***

Total	5133	1276	994	414

Two hundred metaphase spreads were used to determine the accuracy of the proposed rules. From these 192 metaphase images, 7817-segmented objects were obtained and classified into four classes. The proposed rules can efficiently and accurately classify the individual chromosome with > 90% accuracy.

### Conclusions

This work presents a method for chromosome classification using key chromosomal image parameters. We found that the area ratio, the rectangle width ratio, the chromosome width ratio, maximum width ratio and height ratio can be used to efficiently classify chromosome objects into four classes. From our experiments, the accuracy of individual with straight shape and skewed individual chromosomes were 99.42% and 90.67% respectively. This study demonstrated that Class-1 and Class-2 of chromosomal images can be used to efficiently and accurately determine quality of the metaphase images. In other words, these classes of chromosome can be utilized to identify analyzable metaphase spreads. The processing time of chromosome classification is crucial for automated systems since the systems need to process large number of images in order to correctly diagnosis a patient. Consequently, chromosome counting, e.g., Down's syndrome screening can greatly benefit from our proposed chromosome classification. In the future, we planned to integrate existing automatic karyotyping algorithms and other chromosome analysis modules, e.g., numerical and structural abnormally detection. The current metaphase selection module was implemented and used in the MetaSel program. Both software (for Windows XP or 7 only) and user manual can be freely downloaded from our website, http://www4a.biotec.or.th/GI/tools/metasel.

## Availability of supporting data

The user manual of the software and some samples of chromosome images supporting the results of this article are available on our website, http://www4a.biotec.or.th/GI/tools/metasel

## Competing interests

The authors declare that they have no competing interests.

## Authors' contributions

RU carried out the implementation of the MetaSel program, participated in the design of the proposed algorithm. AI, SK and ST analyzed the results and revised the draft of metaphase selection algorithm. PY, RP and AA participated in designing the user interface of the MetaSel program. RU and AI performed experiments and statistical analysis of this work. SK and ST drafted the manuscript. ST conceived the original idea and supervised the production of this work.

## Declarations

The Publication of this article was funded by TRF grant no. RSA5480026 and the funding from National Center for Genetic Engineering and Biotechnology (BIOTEC).

This article has been published as part of *BMC Bioinformatics *Volume 14 Supplement 16, 2013: Twelfth International Conference on Bioinformatics (InCoB2013): Bioinformatics. The full contents of the supplement are available online at http://www.biomedcentral.com/bmcbioinformatics/supplements/14/S16.
